# *Wolbachia w*AlbB remains stable in *Aedes aegypti* over 15 years but exhibits genetic background-dependent variation in virus blocking

**DOI:** 10.1093/pnasnexus/pgac203

**Published:** 2022-09-22

**Authors:** Xiao Liang, Cheong Huat Tan, Qiang Sun, Meichun Zhang, Pei Sze Jeslyn Wong, Meizhi Irene Li, Keng Wai Mak, Abdiel Martín-Park, Yamili Contreras-Perera, Henry Puerta-Guardo, Pablo Manrique-Saide, Lee Ching Ng, Zhiyong Xi

**Affiliations:** Department of Microbiology and Molecular Genetics, Michigan State University, East Lansing, MI 48824, USA; Environmental Health Institute, National Environment Agency, Singapore 138667; Department of Microbiology and Molecular Genetics, Michigan State University, East Lansing, MI 48824, USA; Department of Microbiology and Molecular Genetics, Michigan State University, East Lansing, MI 48824, USA; Environmental Health Institute, National Environment Agency, Singapore 138667; Environmental Health Institute, National Environment Agency, Singapore 138667; Environmental Health Institute, National Environment Agency, Singapore 138667; Laboratorio para el Control Biologico de Aedes aegypti (LCB-UADY), Unidad Colaborativa para Bioensayos Entomologicos, Campus de Ciencias Biologicas y Agropecuarias, Universidad Autonoma de Yucatan, Mérida, Yucatán CP 97315, Mexico; Laboratorio para el Control Biologico de Aedes aegypti (LCB-UADY), Unidad Colaborativa para Bioensayos Entomologicos, Campus de Ciencias Biologicas y Agropecuarias, Universidad Autonoma de Yucatan, Mérida, Yucatán CP 97315, Mexico; Laboratorio para el Control Biologico de Aedes aegypti (LCB-UADY), Unidad Colaborativa para Bioensayos Entomologicos, Campus de Ciencias Biologicas y Agropecuarias, Universidad Autonoma de Yucatan, Mérida, Yucatán CP 97315, Mexico; Laboratorio para el Control Biologico de Aedes aegypti (LCB-UADY), Unidad Colaborativa para Bioensayos Entomologicos, Campus de Ciencias Biologicas y Agropecuarias, Universidad Autonoma de Yucatan, Mérida, Yucatán CP 97315, Mexico; Environmental Health Institute, National Environment Agency, Singapore 138667; School of Biological Sciences, Nanyang Technological Institute, Singapore 637551; Department of Microbiology and Molecular Genetics, Michigan State University, East Lansing, MI 48824, USA

## Abstract

The ability of the maternally transmitted endosymbiotic bacterium *Wolbachia* to induce cytoplasmic incompatibility (CI) and virus blocking makes it a promising weapon for combatting mosquito-borne diseases through either suppression or replacement of wild-type populations. Recent field trials show that both approaches significantly reduce the incidence of dengue fever in humans. However, new questions emerge about how *Wolbachia*-mosquito associations will co-evolve over time and whether *Wolbachia*-mediated virus blocking will be affected by the genetic diversity of mosquitoes and arboviruses in the real world. Here, we have compared the *Wolbachia* density and CI expression of two *w*AlbB-infected *Aedes aegypti* lines transinfected 15 years apart. We have also assessed *w*AlbB-mediated virus blocking against dengue (DENV), Zika (ZIKV), and Chikungunya (CHIKV) viruses and examined whether host genetic backgrounds modulate viral blocking effects by comparing ZIKV infection in mosquitoes with a Mexican genetic background to those with a Singaporean background. Our results show that over 15 years, *w*AlbB maintained the capacity to form a stable association with *Ae. aegypti* in terms of both density and CI expression. There were variations in *w*AlbB-induced virus blocking against CHIKV, DENV, and ZIKV, and higher inhibitory effects on ZIKV in mosquitoes on the Singaporean genetic background than on the Mexican background. These results provide important information concerning the robustness and long-term stability of *Wolbachia* as a biocontrol agent for arbovirus disease control.

Significance Statement The successful implementation of *Wolbachia* for combating mosquito-borne arbovirus diseases relies on a stability of artificial *Wolbachia* transinfection in target mosquito vectors, with a high virus blocking induced in mosquitoes on various genetic backgrounds across different geographic populations. The present studies showed that the *Wolbachia w*AlbB strain maintained a stable association with *Ae. aegypti* over 15 years and virus blocking effects varied among different arboviruses and host genetic backgrounds. Given the ongoing *Wolbachia* release in multiple countries, these results provide important information to guide the development of optimal *Wolbachia* release strategies for disease control, highlighting a potential for global deployment of *Wolbachia* in a region and context specific manner.

## Introduction

Both the distribution range of mosquito vectors and the prevalence of mosquito-borne diseases have rapidly increased because of global warming in recent decades (1–3). A highly effective, sustainable, and environmentally-friendly vector control strategy is urgently needed because of the insufficiency of traditional approaches. Recently, significant efforts have been made to develop *Wolbachia*-based approaches to either reduce the mosquito’s ability to transmit pathogens through population replacement or to suppress the mosquito density below the epidemic risk threshold through population suppression (4–8). Successful field trials have shown that *Wolbachia*-based population replacement has reduced dengue incidence by 77.1% and hospitalization by 86.2% in Indonesia, among several other countries (4, 7, 9), and population suppression has been shown to produce strong suppression or even elimination of *Aedes* mosquito vectors in target areas in multiple countries, with dengue incidence being reduced by 71% to 88% in Singapore (5, 8, 10–12).

Estimated to infect more than 65% of all insect species, *Wolbachia* is a maternally transmitted endosymbiotic bacterium belonging to the order Rickettsiales (13). It is known for its ability to induce cytoplasmic incompatibility (CI), a phenomenon involving early embryonic death that occurs when the *Wolbachia*-infected male mates with either an uninfected female or a female carrying a different strain of *Wolbachia* (14). Based on CI, a conditional sterility can be induced in the field by releasing the incompatible males to mate with naturally uninfected wild-type females, resulting in population suppression. CI also provides a reproductive advantage to *Wolbachia*-infected females as compared to uninfected females, since infected females can produce infected offspring after mating with both infected and uninfected males, whereas uninfected females can reproduce only if they mate with uninfected males. With *Wolbachia* frequencies surpassing a critical equilibrium determined by fitness costs (15), CI would facilitate the invasion and spread of *Wolbachia* into uninfected populations and eventually causes the population infected at high frequency, triggering population replacement (16–18).

Multiple *Wolbachia* strains are able to induce pathogen blocking in mosquitoes (19–22), thus enabling population replacement to reduce pathogen transmission between mosquitoes and humans. *Aedes aegypti*, the primary dengue vector, does not carry the native *Wolbachia* infection (23), whereas *Ae. albopictus*, another important vector of arboviruses, is naturally infected by *Wolbachia* (24). Both can be transinfected by transfer of *Wolbachia* strains from other insect hosts via embryonic microinjection (8, 17). A hallmark of successful transinfection in mosquitoes is a stable maternal transmission of *Wolbachia* at 100% efficiency, a property critical for maintaining a high *Wolbachia* infection frequency in the field, and for quality production of *Wolbachia*-infected male mosquitoes for the suppression approach. In addition to maintaining the ability to induce CI, artificial *Wolbachia* infection in transinfected *Ae. aegypti* can inhibit a variety of arboviruses, including dengue (DENV), Zika (ZIKV), and Chikungunya (CHIKV) viruses (20). Evidence indicates that the strength of the *Wolbachia*-mediated viral interference is often correlated with the density of *Wolbachia* in the somatic tissues, such as the midguts and salivary glands, of transinfected mosquitoes (25). Although not yet fully understood in this context, immune priming and altered metabolism are the two physiological changes that could contribute to the underlying mechanism of *Wolbachia*-mediated viral blocking (26, 27). Depending on the *Wolbachia* strain, a transinfected mosquito can show strong, moderate, or no resistance to arboviruses (28–30). While the *w*Mel strain, a complete CI inducer in transinfected mosquito but weak CI inducer in its original *Drosophila* host (31, 32), has been successfully demonstrated to reduce dengue incidence in field trial, evidence indicates that *w*AlbB and *w*MelCS outperform *w*Mel in reducing the viral transmission potential according to viral loads in the mosquito saliva (21). The strength of *Wolbachia*-mediated viral inhibition can also be affected by both the host and viral genetic backgrounds. Multigenerational artificial selection with regard to pathogen blocking for DENV in *w*Mel-infected *Ae. aegypti* has resulted in a significant divergence of mosquito populations with either low or high virus blocking, which is associated with single nucleotide polymorphisms of mosquito genes on chromosome 1 (33). *Wolbachia*-mediated viral blocking has been found to vary among the four DENV serotypes, with inhibition of DENV-1 being consistently less effective than the others (21, 30, 34, 35). Furthermore, evidence indicates global genetic diversity of *Ae. aegypti* and geographic variation in vector competence (36, 37), likely contributing to the variation of *Wolbachia*-mediated blocking in these mosquitoes. Accordingly, it is unclear how the efficacy of disease control will be affected by variation in *Wolbachia*-mediated viral blocking resulting from genetic diversity in mosquitoes and arboviruses in the real world that *Wolbachia* will encounter when deployed across a global landscape in the future. These emerging pieces of evidence call for an in-depth characterization of the impact of host and virus genomes on viral blocking, knowledge that is essential for widespread use of the replacement strategy.

As the first *Wolbachia* strain established in both *Ae. aegypti* and *Anopheles* mosquitoes, *w*AlbB induces complete CI, as observed in its original host, *Ae. albopictus* (38), and strong pathogen blocking in transinfected lines (17, 19, 39, 40). Recent studies have shown that *w*AlbB is more stable at high temperature than *w*Mel in *Ae. aegypti*, leading to the proposed release of *w*AlbB in areas where it is challenging for *w*Mel to establish infection because of its high susceptibility to extreme summer temperatures and wide yearly temperature ranges (41–43). Based on analysis of the *w*AlbB whole-genome sequence, only four single nucleotide variants have occurred over 15 years of transinfection (43). A benefit of this stable performance is that *w*AlbB-infected *Ae. aegypti* have been effectively mass-reared to produce incompatible males for release into the field for population suppression in the United States, Singapore, Australia, and Mexico (5, 6, 11, 12, 44). Release of *w*AlbB-infected *Ae. aegypti* for population replacement has also resulted in a significant reduction in dengue incidence in Malaysia (45). After being introduced into various mosquito genetic backgrounds through outcrosses, *w*AlbB maintains a stable association with *Ae. aegypti* (43, 45–47). However, one study has shown that the impact of *w*AlbB on mosquito life traits depends on the host’s genetic background (47), whereas another study has shown that the effects are consistent across two mosquito backgrounds (43). While a recent study provides extensive data showing a decade of stability for *w*Mel in the field after release (48), evidence has also been accumulating over the last few years for *w*AlbB to explore the long-term stability of the transinfection for disease control (43). Questions remain to fully explore are whether the titers of the artificial *Wolbachia* infections will attenuate over time and, if so, how quickly that attenuation will occur, resulting in a breakdown of mediated viral blocking or CI expression. One way which has not yet been done to address them is direct comparisons between old lines and ones with a more novel association.

We developed the first *w*AlbB-infected *Ae. aegypti* line, WB1, in 2005 (17). Fifteen years later, to test the stability of the *w*AlbB–*Ae. aegypti* association, we repeated this transinfection assay to introduce *w*AlbB from *Ae. albopictus* into *Ae. aegypti* and generate the WB2 line. Phenotypic comparisons between WB1 and WB2 have now demonstrated that over the course of those 15 years, *w*AlbB maintained the capacity to have a stable density and perfect maternal transmission efficiency and induce strong CI in the natural Waco genetic background. We also found that *w*AlbB induced a significant inhibitory effect against DENV, ZIKV, and CHIKV in *Ae. aegypti*. In addition, *w*AlbB-infected *Ae. aegypti* with a Singaporean genetic background exhibited a stronger viral blocking than did those on a Mexican genetic background, indicating an impact of mosquito host genetic background on *Wolbachia*-mediated virus blocking. These results demonstrate the stability and robustness of *w*AlbB transinfection in *Ae. aegypti*, with the maintenance of properties critical for the suppression of the *Ae. aegypti* mosquito population and viral blocking ability for reducing arbovirus transmission.

## Results

### Generation of the *Ae. aegypti* WB2 line with a *w*AlbB infection

To examine the long-term stability of *w*AlbB in *Ae. aegypti*, we generated an identical *w*AlbB transinfection in *Ae. aegypti* to compare it with the WB1 line, which was established in 2005 and had the US (Waco, TX, USA) genetic background (17). The wild-type *Ae. albopictus* Houston (HOU) line was used as the donor, and the cytoplasm of each individual HOU embryo was transferred to wild-type *Ae. aegypti* by embryogenic microinjection. Experiments were repeated three times, in each case with approximately 200 to 400 embryos injected. Females (G0) developing from the embryos that survived the microinjection were mated with wild-type males (Waco), then allowed to blood-feed. After their offspring (G1) were produced, the G0 females were sacrificed and screened for *Wolbachia* infection by PCR assay. In the third experiment, three of the five surviving females were positive for *w*AlbB infection (Fig. S1a). Only the progeny from the positive females were selected for outcross with Waco males to produce the next generation. Six of the 21 G1 isofemales (28.5%) were observed to carry the *w*AlbB infection. At G2, 16 of 45 females had *w*AlbB infections. From them, seven females that showed strong infections were selected to establish the next generation (G3). The subsequent PCR assay showed that all the tested G3 individuals (*n* = 10) carried *w*AlbB. This transinfected line is hereafter referred to as the WB2 line. Subsequently, we randomly selected 10 to 20 individuals from G4 to G8 for PCR assay, and all were positive for *w*AlbB, indicating 100% maternal transmission efficiency (Fig. S1b). Although initial egg hatch rates were low (29% to 50%), they recovered to a level close to 70% from G5 onward (Fig. S1b), likely thanks to the removal of inbreeding effects through repeated outcrosses of WB2 females with Waco males. This new transinfected line provided us the opportunity to study phenotypic effect of *w*AlbB on hosts over time.

### No difference in CI induction between the WB1 and WB2 lines

**Fig. 1.**
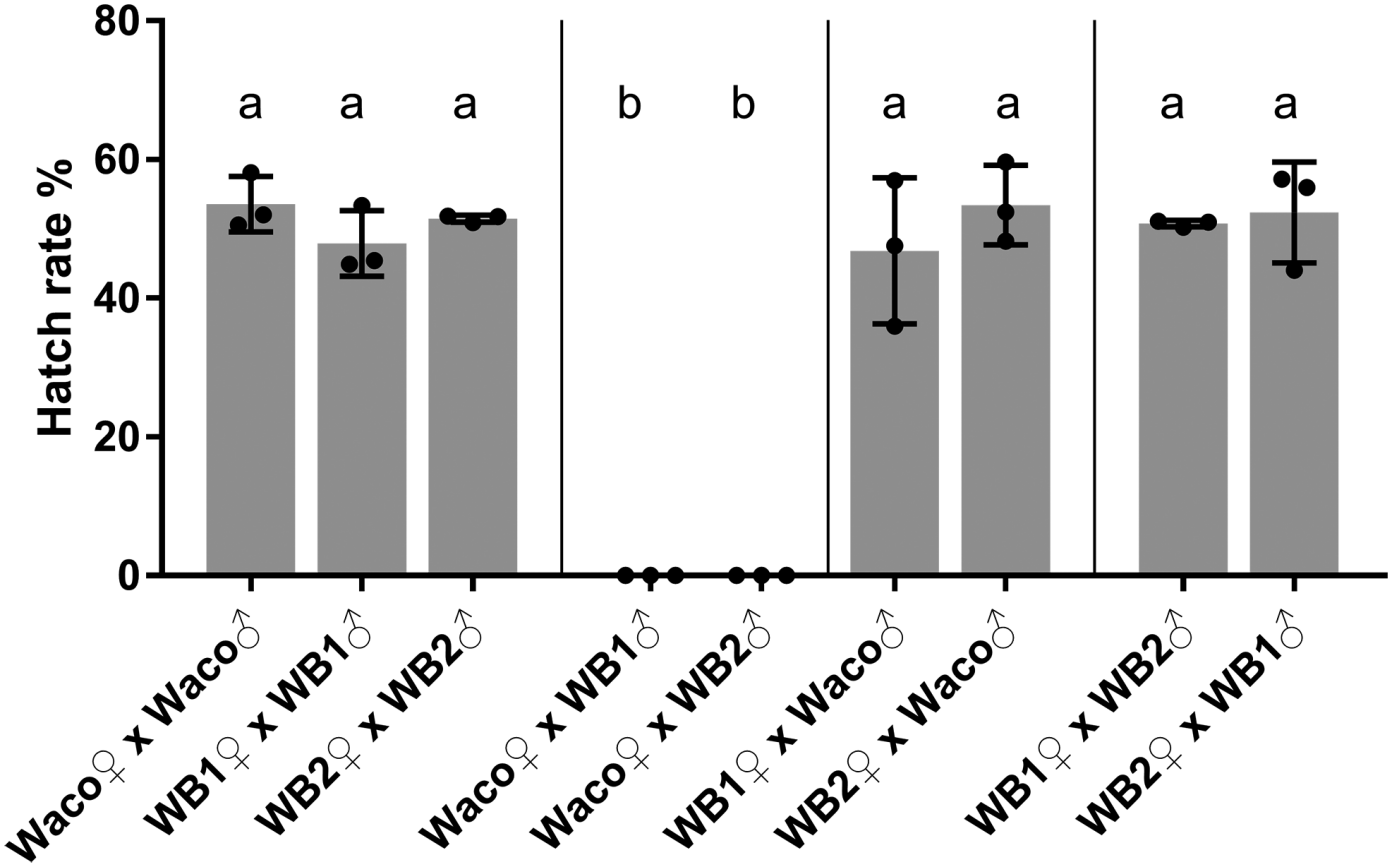
CI crosses involving wild-type Waco, WB1, and WB2 mosquitoes. The results are expressed as the mean of three replicates for each cross involving 10 females and 10 males. Dots indicate the sample values. Error bars indicate the SD. The letters above the columns indicate significant differences: *P* < 0.0001 by ANOVA-Tukey’s multiple comparison test.

To determine whether there was any difference in *w*AlbB-induced CI between the WB1 and WB2 lines, we set up crosses involving the WB1, WB2, and Waco lines. The self-crosses of WB1, WB2, and Waco yielded hatch rates of 47.9% (95% interval = 36.1% to 59.7%), 51.5% (95% interval = 50.2% to 52.8%), and 53.6% (95% interval = 43.6% to 63.5%), respectively. The WB1 males were compatible with WB2 females, with an average hatch rate of 52.4% (95% interval = 34.3% to 70.4%). Similarly, WB2 males were also compatible with WB1 females, with a hatch rate of 50.8% (95% interval = 49.6% to 52.0%). Both WB1 and WB2 males induced a 100% CI when crossed with Waco females (95% interval = 0% to 0%) (Fig. 1). The results indicate that WB1 and WB2 are compatible with each other but incompatible with wild-type *Ae. aegypti*, pointing to lack of host effects on *w*AlbB-induced CI after its transfer into *Ae. aegypti* for 15 years (or approximately 180 generations).

### No difference in *w*AlbB density in female whole bodies and male testes between the WB1 and WB2 lines

**Fig. 2.**
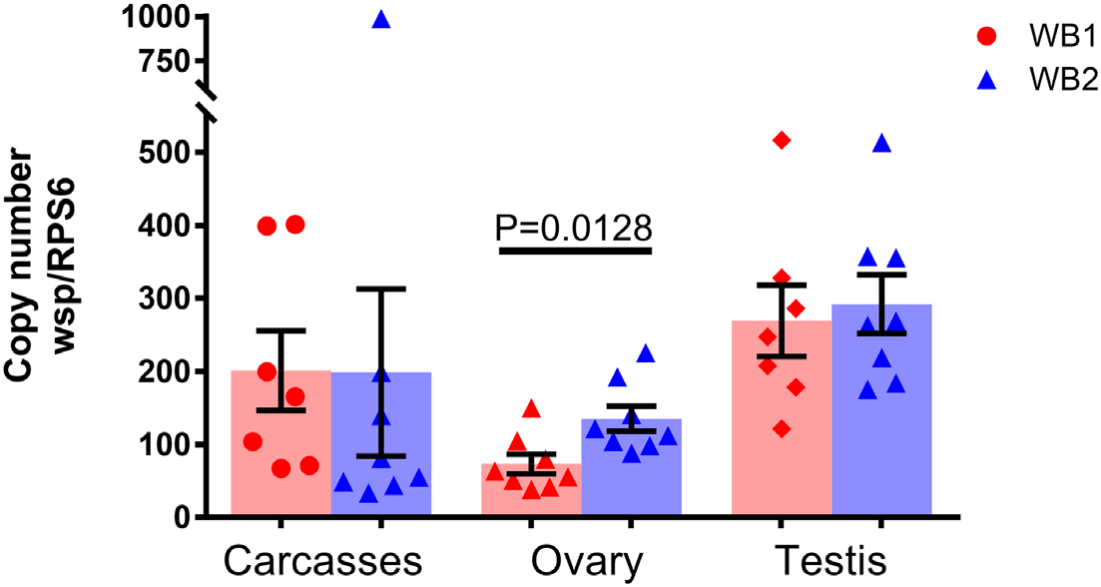
The density of *w*AlbB in WB1 and WB2 mosquitoes. The copy number of the *Wolbachia wsp* gene was normalized by the mosquito *rps6* gene. Each point represents an individual tissue. Data are shown as the mean of eight replicates ± SE. Error bars indicate the SE. *P* = 0.0128, Student’s *t*-test.

To examine the impact of long-term association with *Ae. aegypti* on the tissue distribution of *w*AlbB in a more natural host, rather than an inbred line, we compared the densities of *w*AlbB in whole bodies and reproductive tissues, including ovaries and testes, between the WB1 and WB2 lines after both were outcrossed with Waco for seven generations to homogenize the host genetic background (Fig. S2). The results showed no difference in *w*AlbB density in the female whole bodies and male testes between the WB1 and WB2 lines. However, WB2 ovaries showed a significantly higher *Wolbachia* density than did the WB1 ovaries (Fig. 2). These results indicate that *w*AlbB maintains a stable density in an outbred genetic background after associated with *Ae. aegypti* at the laboratory conditions for 15 years, which is consistent with a recent report showing a stable *w*AlbB in transinfected *Ae. aegypti* under two different host backgrounds (43).

### Variation among different arboviruses in the strength of virus blocking by *w*AlbB

**Fig. 3.**
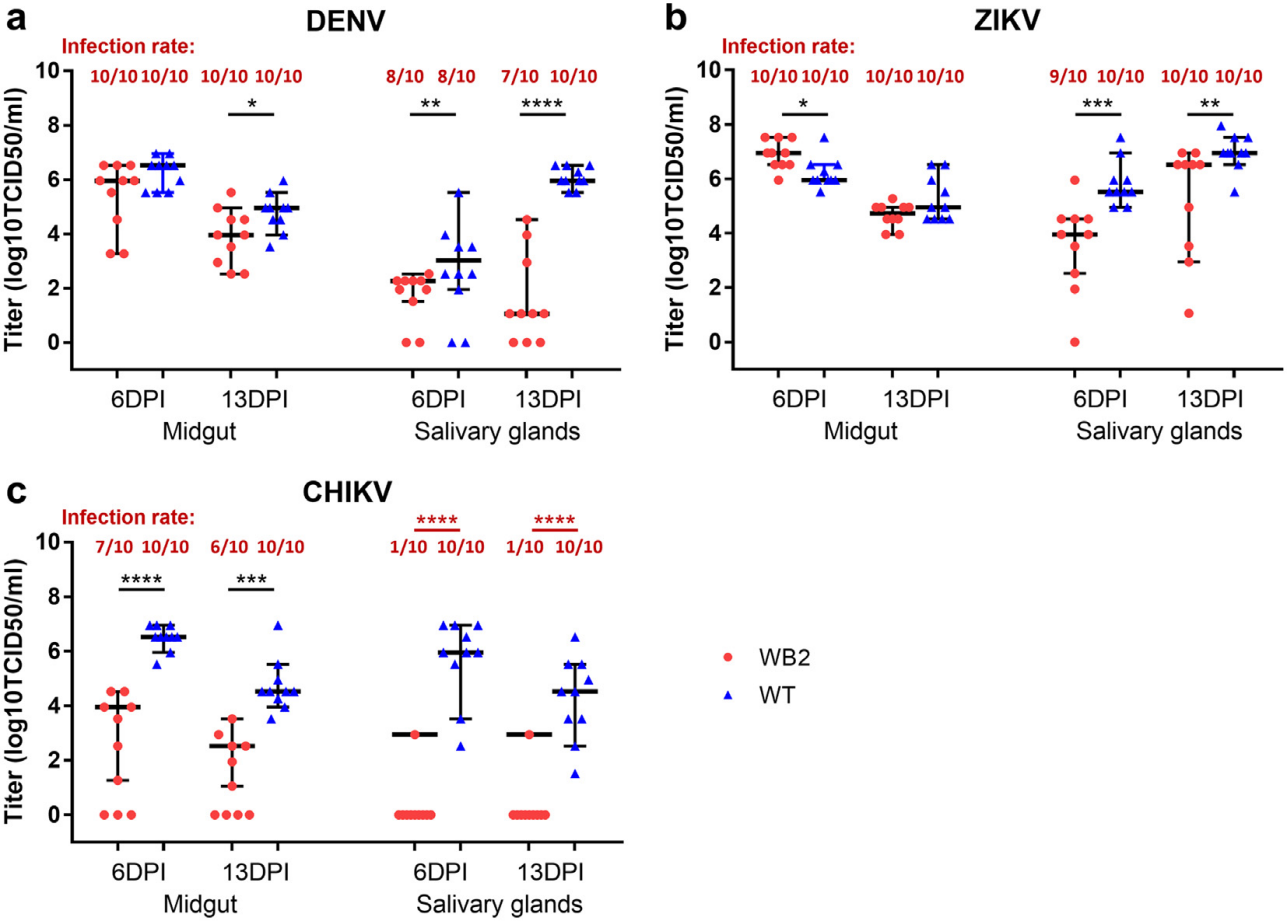
Vector competence for DENV, ZIKV, and CHIKV of *w*AlbB-infected *Ae. aegypti* on the Singaporean genetic background. After the outcrossed WB2 and wild-type (WT) mosquitoes, both on a Singaporean genetic background, had been fed with blood spiked with either DENV-2 at 7.74 log_10_TCID_50_/ml (a), ZIKV (MR766 strain) at 8.74 log_10_TCID_50_/ml (b), or CHIKV (EHIKJ71albY08 strain) at 6.24 log_10_TCID_50_/ml (c), the midguts and salivary glands were collected at 6 and 13 DPI and assayed for viral infection. Virus infection levels were determined using a viral titration assay and expressed as log10TCID50/ml. Lines and error bars denote medians ± 95% CIs of viral titers with nonzero values, and each point represents an individual midgut/salivary gland. Significant differences in viral titers and infection rate (prevalence) were determined using Mann–Whitney test and two-sided Fisher’s exact test, respectively. *P < 0.05, **P < 0.01, ***P < 0.001, ****P < 0.0001.

Vector competence assays were performed to measure the *w*AlbB-mediated blocking effects on DENV, CHIKV, and ZIKV in *Ae. aegypti* with a Singaporean genetic background. After mosquitoes were fed with an infectious bloodmeal, midguts and salivary glands were collected at 6 and 13 d post-infection (DPI) to measure viral titers and infection rates. We observed 100% infection rates in the midguts and salivary glands of wild-type mosquitoes in all the infection assays with the three viruses, except for an 80% DENV infection rate in the salivary glands at 6 DPI (Fig. 3). In the assays with DENV serotype 2 (DENV-2) at 6 DPI, *w*AlbB significantly inhibited viral loads in the salivary glands (95% interval = 1.87 to 2.39) as compared to the wild-type mosquitoes (95% interval = 2.30 to 4.21, *P* = 0.0057), whereas both WB2 and wild-type had a similar viral loads in the midgut (*P* = 0.13). At 13 DPI, *w*AlbB significantly inhibited viral loads in both midguts (95% interval = 3.16 to 4.62) and salivary glands (95% interval = 0.81 to 3.66) as compared to their wild-type counterparts (midguts, 95% interval = 4.28 to 5.28, *P* = 0.049; salivary glands, 95% interval = 5.79 to 6.34, *P* < 0.0001) (Fig. 3a). In the ZIKV infection assays, although the salivary gland infection rates were about the same for wild-type and WB2 mosquitoes, the viral titers in the salivary glands of the WB2 mosquitoes were significantly reduced at both 6 (95% interval = 3.02 to 4.84) and 13 DPI (95% interval = 3.77 to 6.72), respectively, when compared to those of wild-type mosquitoes (6 DPI, 95% interval = 5.25 to 6.42, *P* = 0.0008; 13 DPI, 95% interval = 6.51 to 7.45, *P* = 0.0044). Although not observed in midguts at 13 DPI, a reduction in viral titers was also observed in the WB2 midguts at 6 DPI (95% interval = 6.52 to 7.27) as compared to the wild-type mosquitoes (95% interval = 5.82 to 6.60, *P* = 0.01) (Fig. 3b). In the CHIKV infection assays of the midguts sampled at both time points, *w*AlbB reduced the viral titers significantly in the WB2 mosquitoes (6 DPI, 95% interval = 2.37 to 4.56; 13 DPI, 95% interval = 1.53 to 3.31) as compared to the wild-type mosquitoes (6 DPI, 95% interval = 6.17 to 6.82, *P* < 0.0001; 13 DPI, 95% interval = 4.05 to 5.40, *P* = 0.0002). In the salivary glands at both time points, *w*AlbB decreased the infection rates by 90% (*P* = 0.0001) (Fig. 3C). These results indicate that *w*AlbB induced resistance to all three arboviruses in *Ae. aegypti*, with the strength of the viral blocking varied among them. Although *w*AlbB-mediated suppression of dengue virus has been reported previously (19) and a suppression of ZIKV was published (40) while this manuscript was in review, this is the first showing suppression of CHIKV by *w*AlbB, indicating its spectrum of antiviral activity similar to *w*Mel (49).

### Variation in the strength of virus blocking by *w*AlbB on different host genetic backgrounds

**Fig. 4.**
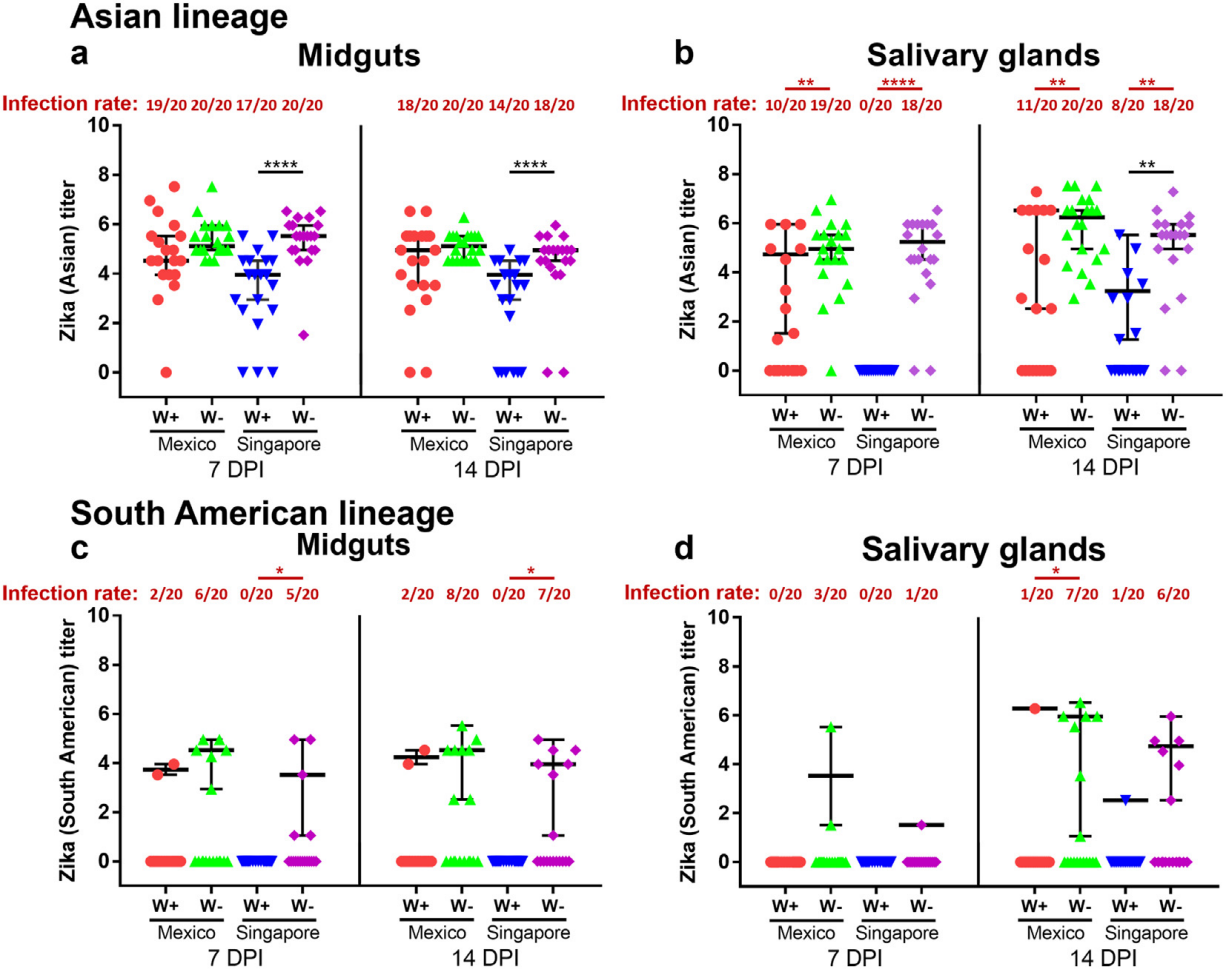
Vector competence of *w*AlbB-infected *Ae. aegypti*, on either the Mexican or Singaporean genetic background, for ZIKV. The infection rates and titers of ZIKV LP0210Y17 strain, an Asian lineage (a, b), and SG(EHI)ZIKV/33164Y17, a South American lineage (c, d), in the midguts (a, c) and salivary glands (b, d) at 7 and 14 DPI. The virus titers were calculated as log10TCID50/ml. W+, *w*AlbB-infected mosquitoes on either the Mexican genetic background (WBM) or Singaporean genetic background (WBSG); W-, wild-type *Ae. aegypti* (*Wolbachia* free) on either the Mexican genetic background (AFM) or Singaporean genetic background (WTSG). Lines and error bars denote medians ± 95% CIs of viral titers with nonzero values, and each point represents an individual midgut/salivary gland. Significant differences in viral titers and infection rate (prevalence) were determined using Mann–Whitney test and two-sided Fisher’s exact test, respectively. *P < 0.05, **P < 0.01, ****P < 0.0001.

We then compared the ability of *w*AlbB to block two ZIKV lineages, an Asian and a South American lineage, in *Ae. aegypti* with genetic backgrounds from Mexico or Singapore. The ZIKV infections were measured in both midguts and salivary glands at 7 and 14 DPI. For both the Mexican and Singaporean mosquito genetic backgrounds, *w*AlbB exhibited virus inhibition at both time points (Fig. 4). For the Asian lineage, virus loads were significantly affected due to *Wolbachia* (generalized linear model [GLM]: *F* = 26.62, *df* = 1, *P* < 0.0001), mosquito genetic background (GLM: *F* = 135.41, *df* = 1, *P* < 0.0001) and their interactions (GLM: *F* = 8.42, *df* = 1, *P* = 0.004). There were significant effects of mosquito genetic background on virus loads in both *w*AlbB-infected midguts and salivary glands (GLM: *F* = 14.05, *df* = 1, *P* = 0.0003), whereas similar effects were not observed in wild-type mosquitoes (GLM: *F* = 1.92, *df* = 1, *P* = 0.17). Indeed, there was a consistent pattern that *w*AlbB-infected *Ae. aegypti* on the Singaporean genetic background induced stronger viral blocking than did those on the Mexican genetic background as described below. For the Singaporean background, virus titers were significantly lower in the midguts of *w*AlbB-infected mosquitoes at both 7 DPI (95% interval = 3.36 to 4.44) and 14 DPI (95% interval = 3.39 to 4.27) than those of wild-type mosquitoes (7 DPI, 95% interval = 4.84 to 5.85, *P* < 0.0001; 14 DPI, 95% interval = 4.59 to 5.13, *P* < 0.0001), respectively. However, a similar difference was not observed in midguts for mosquitoes on the Mexican background at both time points (Fig 4a). In salivary glands at 7 DPI, a complete virus blocking was observed in *w*AlbB-infected mosquitoes on the Singaporean background as compared to a 90% infection rate in wild-type mosquitoes (*P* < 0.0001), whereas *w*AlbB induced partial virus blocking, with a 50% infection rate, on the Mexican background as compared to a 95% infection rate in the wild-type mosquitoes (*P* = 0.003) (Fig. 4b). In salivary glands at 14 DPI, virus infection rates significantly decreased in *w*AlbB-infected mosquito on both genetic backgrounds as compared to wild-type mosquitoes (Singaporean, *P* = 0.002; Mexican, *P* = 0.001). While similar effects were not observed in mosquitoes on the Mexican background, *w*AlbB significantly decreased the median viral titers by 2.29 log_10_TCID_50_/ml in mosquitoes on the Singaporean background (95% interval = 2.08 to 4.58) as compared to their *Wolbachia*-free counterparts (95% interval = 4.81 to 5.95, *P* = 0.002) (Fig. 4b). For the South American lineage, a complete virus blocking was observed in the WB2 midguts on the Singaporean backgrounds at both 7 and 14 DPI as compared to their *Wolbachia*-free counterparts (7 DPI, *P* = 0.047; 14 DPI, *P* = 0.0083) (Fig. 4c), perhaps because of the low viral titer in the infectious bloodmeal, whereas only partial virus blocking occurred in salivary glands of mosquitoes on the Mexican background at 14 DPI (*P* = 0.043) (Fig. 4d). These results demonstrate that the host’s genetic background can modulate the strength of *w*AlbB-induced virus blocking in *Ae. aegypti*, which is consistent with the previous studies showing that variations of mosquito genes affect *w*Mel-mediated dengue blocking (33).

### Variation in mating competitiveness between WB2 and wild-type males on the Mexico genetic background

As an effort to develop a WB2 mosquito line for population suppression in Mexico (12, 44), male mating competitiveness assays were carried out to measure the ability of the outcrossed WB2 (WBM) males on the Mexican genetic background to compete with the Mexican wild-type (AFM) males for mating with wild-type females. Five cages containing different ratios of WBM males to AFM males to AFM females (0:1:1, 1:0:1, 1:1:1, 5:1:1 and 10:1:1) were set up. As expected, when only WBM males were present, none of eggs hatched (Table 1). In the cage with the ratio of 5:1:1, the observed egg hatch rate was not significantly different from the expected values assuming an equal competitiveness of WBM and AFM males and random mating (*P* = 0.12). For the other two ratios, however, the observed egg hatch rates were higher than the expected values (1:1:1, *P* = 0.02; 10:1:1, *P* = 0.01). These results indicate that the mating competitiveness of WB2 males on the Mexican genetic background varies by ratios and may be slightly reduced compared to wild-type males, which is consistent with the previous observation of mating competitiveness of WB2 males on two different genetic backgrounds (47).

**Table 1.** Mating competitiveness of *Ae. aegypti* males on the Mexican genetic background at various release ratios.

**WBM♂:AFM♂:AFM♀**	**Number of eggs**	**Observed egg hatch rate**	**Expected egg hatch rate**	**Competitiveness index**	*P* -value*
0:1:1	303	0.77			
1:0:1	331	0.00 (0)	0.00		>0.99
1:1:1	1,071	0.42 (449)	0.39	0.84	0.02
5:1:1	494	0.10 (51)	0.13	1.30	0.12
10:1:1	340	0.11 (36)	0.07	0.63	0.01

*Comparisons between the observed and expected egg hatch through exact binomial test. WBM: outcrossed *w*AlbB-infected *Ae. aegypti* on the Mexican genetic background; AFM: wild type *Wolbachia*-free *Ae. aegypti* on the Mexican genetic background.

## Discussion

The success of the *Wolbachia* technology in suppressing an *Ae. aegypti* mosquito population or reducing its ability to transmit arboviruses hinges on the long-term stability of the *Wolbachia* infection, the *Wolbachia*-induced antiviral properties, and strong CI expression. In this work, we have demonstrated the successful establishment of another *w*AlbB-infected *Ae. aegypti* WB2 line with a stable 100% maternal transmission efficiency. Comparison of the WB2 line to the WB1 line, generated 15 years ago, has demonstrated a long-term stability of the *w*AlbB-*Ae. aegypti* association, with neither alteration of CI expression nor attenuation of the *Wolbachia* titer in female whole bodies and male testes. *w*AlbB induced resistance to DENV, ZIKV, and CHIKV. In addition, it conferred a strong resistance to both South American and Asian lineages of ZIKV in WB2 mosquitoes on either a Mexican or Singaporean genetic background. We also found that the outcrossed WB2 males on the Mexican genetic background had a mating competitiveness that was comparable to that of their counterpart wild-type males. These results support the feasibility of developing *w*AlbB-based population replacement and suppression strategies for controlling DENV, ZIKV, and CHIKV in disease-endemic countries.

Control of mosquito-borne diseases through *Wolbachia*-based population replacement relies on the long-term stability of the *Wolbachia*-mediated viral blocking properties in the transinfected line, in which the *Wolbachia*-mosquito association may co-evolve over time. Establishment of the WB2 line has enabled us to examine the stability of artificial *w*AlbB infection in *Ae. aegypti* over 15 years. We previously discovered that the density of the native *w*AlbB in the donor species *Ae. albopictus* is much lower than that in the transinfected WB1 mosquitoes, raising a concern that the titer of *w*AlbB may become attenuated after co-adaptation occurs between the symbiont and its host over a long period of time (25). Since the density of *Wolbachia* is often associated with the strength of its induced viral inhibition, such attenuation could result in the reduction or even loss of its viral-blocking properties (25). Our results show that, in an outbred genetic background, WB1 and WB2 had similar densities in both female whole bodies and male testes except for in the ovaries, where WB2 had higher density than did WB1. A similar observation was made in a recent study that compared the density of *w*Mel between a recently transinfected line and another line generated 10 year ago (50). It is predicated that CI genes would degrade over time in selectively neutral populations, starting with the sperm modification factor *cifB*, followed by the rescue factor *cifA* (51). The fact that *w*Mel induces weak CI in *Drosophila melanogaster* but complete CI in mosquito also provides a direct evidence of a strong host effect that supports the existence of segregating host suppressors in natural hosts. We observed that WB1 and WB2 were bidirectionally compatible and induced the same CI pattern, indicating that the phenotypic expression of CI factors remained the same, which is different from natural systems (e.g. *D. melanogaster* and *D. yakuba*) where host factors are known to modify CI strength (52, 53). These results indicate that *w*AlbB transinfection has been stable for >15 years under laboratory conditions, consistent with a recent report showing few genetic changes occurring during this period (43). Although it may eventually evolve to a low density as observed for *Ae. albopictus*, this decrease probably will be a long-term process and should not affect the efficacy of disease control through viral blocking in the short- or middle-term. This is supported by a recent study showing that *w*AlbB maintains high density and dengue inhibition at least over 1 year after introduced into the field (54). Further studies are needed to determine whether real-world field conditions will facilitate the co-adaptation of *w*AlbB and *Ae. aegypti* to accelerate the process. It is worthy to note that we have compared *Wolbachia* densities between WB1 and WB2 after outbred with Waco because such an outcross with a local genetic background is the first step when a transinfected line is deployed to a country/region for disease control. Evaluation of *w*AlbB’s capacity to form a stable association in the outbred line would provide more direct implication for implementation than that in the inbred line. However, a comparison of WB2 with the inbred WB1 line through reciprocal crosses would allow to examine how *w*AlbB and *Ae. aegypti* differentiate during the co-adaptation.

In our study, *w*AlbB was able to limit CHIKV, DENV, and ZIKV replication and/or dissemination in transinfected *Ae. aegypti* when compared to wild-type mosquitoes. In wild-type mosquitoes, the infection rates were 100% for all three viruses in the midguts and salivary glands at 6 and 13 DPI, respectively, indicating that the infection doses were sufficient to develop transmissible infection in the mosquitoes. In comparison to wild-type mosquitoes, CHIKV infection rates in WB2 mosquitoes were reduced by 90% in the salivary glands at both 6 and 13 DPI. For DENV-2 and ZIKV, viral titers were significantly lower in WB2 salivary glands at both time points as compared to their wild-type counterparts, but infection rates were not statistically significant. This variation in virus blocking is probably a result of differences in their viral genomes, life cycles, and required host factors between the alphavirus (CHIKV) and the flaviviruses (DENV and ZIKV). Previous studies also observed variations in *Wolbachia*-mediated virus blocking across different DENV serotypes with underlined mechanisms unclear (21, 30, 34). We cannot rule out the possibility that differences in the infectious bloodmeal viral titers may have contributed to the degree or magnitude of the blocking effects observed. Our results also show that viral inhibition was much stronger in the salivary glands than in the midguts, highlighting the potential for underestimating the *Wolbachia*-mediated virus blocking effect if a viral assay is only conducted on midguts.

It has been shown that the genetic variations among different geographic *Ae. aegypti* populations can influence their competence to transmit various viral pathogens (55). Previous studies have also shown that genetic variation in mosquitoes affects *w*Mel-mediated dengue blocking (33). Therefore, it is expected that the host genetic background may also influence the strength of the *w*AlbB-mediated inhibition of ZIKV. In the present study, we observed a consistent pattern that *w*AlbB induced a stronger inhibition of the Asian lineage of ZIKV in mosquitoes on the Singaporean genetic background than in those on the Mexican genetic background. It is unknown whether this variation is caused by a direct impact on viral interference or an indirect impact on the modulation of *Wolbachia* density. A previous study in *Drosophila* has reported that the *Wolbachia* genome has a much greater influence on the level of antiviral protection than does the host genome and that it is *Wolbachia* rather than the host that controls the *Wolbachia* density (56). In our study, the hosts’ genetic background may have played a significant role in the *w*AlbB-mediated viral blocking effect, since the replication and dissemination of both ZIKV lineages were, overall, suppressed to much lower levels in the WB2 mosquitoes on the Singaporean genetic background than in those on the Mexican genetic background. Consistent with our results, a recent study has reported that *w*AlbB inhibits ZIKV in *Ae. aegypti* on an Australian background, a line that was also derived from WB2 through outcrosses (40); interestingly, the authors used a Zika virus strain from Brazil for their vector competence assay and found that *w*AlbB reduced the ZIKV prevalence in saliva by 6- to 7-fold, a similar level of inhibition of ZIKV with a South America lineage in salivary glands that we observed here.

The discovery of variation in *Wolbachia*-mediated viral blocking among various arboviruses and various host genetic backgrounds highlights a caution with regard to deploying *Wolbachia* for disease control in endemic countries in which it will encounter diversity in mosquitoes and arboviruses. The same *Wolbachia* strain may have a high efficacy in reducing viral transmission in one country but produce a different outcome in another country because of differences in terms of local mosquitoes’ genetic background and locally circulating viruses. It is likely that the *Wolbachia*-mediated virus-blocking effect can also be affected by differences in the environmental conditions into which the *Wolbachia*-infected mosquitoes are released. In *Drosophila*, cool temperature is reported to reduce the native *w*Mel oocyte abundance and maternal transmission and this temperature-dependent transmission can explain the fluctuation of continent-wide *w*Mel frequency (57). While some strains are maintained at high, stable equilibria, such as *w*Ri in *D. simulans* (58), host-*w*Mel combinations from the temperate produced a higher rate of transmission in the cold that than tropical genotypes (57). Accordingly, an impact of environmental conditions on maternal transmission, CI, and invasion of *Wolbachia* into field populations has recently been documented in transinfected *Ae. aegypti* (59, 60). The high sensitivity of *w*Mel to heat stress and other factors makes the transinfected mosquito difficult to establish in some areas in Vietnam and Brazil, where *w*AlbB and a heat-resistant *w*MelM variant have been proposed as an alternative because of their stability in extreme temperatures (41, 50, 61, 62). When *Wolbachia* is to be deployed globally for arbovirus disease control, different *Wolbachia* strains may be required for regions with specific environmental or ecological conditions. Thus, a comprehensive profile of various *Wolbachia* strains on the local mosquito genetic background should facilitate and guide future implementation of *Wolbachia* for arbovirus disease control in various field settings. It is expected that such a profile would include an evaluation of the stability of the *Wolbachia* infection under various environmental conditions and with various levels of maternal transmission, CI expression, and pathogen blocking against contemporary viruses.

Since it was generated, the WB2 line has been outcrossed to local *Ae. aegypti* from Singapore, Mexico, and Australia for population suppression field trials (5, 11, 44), and WB1 was released in the United States for the same purpose (6, 63). The slightly reduced male mating competitiveness (index values ranging from 0.63 to 1.30) of WB2 on a Mexican genetic background that we describe here is similar to what was found in a previous study using WB2 outcrossed with a different Mexican mosquito population (index values ranging from 0.57 to 0.79) (47) and is also consistent with the high performance of released males and successful population suppression observed under both semifield and field conditions (5, 6, 11, 46, 64). The WB2 line outcrossed with Singaporean *Ae. aegypti* mosquitoes also showed comparable mating competitiveness with local *Ae. aegypti* mosquitoes and demonstrated for the first time that the *Wolbachia*-based suppression approach is able to reduce the wild-type *Ae. aegypti* mosquito population in an urban landscape (11).

In addition to comparable mating competitiveness with wild-type males, the success of population suppression strategy requires for the stability of *Wolbachia* infection and perfect maternal transmission that are critical for large-scale production of male *Wolbachia*-infected mosquitoes for release. Results from the present study show that *w*AlbB can retain complete CI, a high density in mosquitoes, and 100% maternal transmission over 15 years. These findings are consistent with results obtained for *w*AlbB that has been introgressed onto a Singaporean genetic background since 2016 and is currently being extensively trialed to evaluate its effectiveness in suppressing *Ae. aegypti* mosquito populations (11). As part of the quality assurance evaluation for large-scale production of male *w*AlbB-infected *Ae. aegypti* for release, regular screening and testing of *w*AlbB have shown that *Wolbachia* infections remain stable, with no loss of the bacterium detected, and there has been complete CI and 100% maternal transmission since Singapore’s Project *Wolbachia* field study started 6 years ago. Ross and colleagues have also demonstrated that *w*AlbB genome is stable with very few changes over 15 years and showed perfect maternal transmission and CI in both Australian and Malaysian host backgrounds (43). These results indicate that releases of *w*AlbB-infected males for suppression of *Ae. aegypti* mosquito populations are likely to remain effective.

In conclusion, we have established a recent *w*AlbB transinfection in *Ae. aegypti* and shown that *w*AlbB can reduce the potential of *Ae. aegypti* to transmit DENV, ZIKV, and CHIKV. Together with perfect maternal transmission, complete CI, and high male mating competitiveness, these results support the feasibility of scaling up WB2 release for controlling arboviruses in Singapore and Mexico, as is currently underway (11, 12). The stability of the *w*AlbB–*Ae. aegypti* association is expected to further boost cost-effectiveness and sustainability of both population replacement and suppression strategies. Further studies should include identifying the key factors affecting the long-term stability of *Wolbachia*-host associations under mass-rearing conditions and in the field in disease-endemic countries.

## Materials and methods

### Mosquito lines and maintenance

The wild-type *Ae. albopictus* HOU line carries a native superinfection of *w*AlbA and *w*AlbB (24). Waco is a wild-type *Ae. aegypti* line that does not carry a native *Wolbachia* infection. WB1 is a *w*AlbB-infected line that was developed previously (17). AFM is a wild-type *Ae. aegypti* line that was recently established in the laboratory using eggs collected in the field in Merida, Mexico. WBM denotes the *w*AlbB-infected *Ae. aegypti* derived by repeated outcrossing of WB2 females with AFM males for seven generations. WTSG is a wild-type *Ae. aegypti* line from Singapore and established as previously described (49), and WBSG is the *w*AlbB-infected *Ae. aegypti* line derived by repeated outcrossing of WB2 females with WTSG mosquitoes for seven generations. All the mosquito lines were maintained on 10% sugar solution at 27 ± 1°C and 80 ± 10% relative humidity (RH), with a 12:12 h light:dark photoperiod, according to standard rearing procedures. For routine colony maintenance and experimental studies, female mosquitoes were provided with sheep or swine blood at day 7 post-eclosion, and eggs were collected at 2-d post-bloodmeal.

### Transinfection to generate the WB2 line

The WB2 line was generated by transferring *w*AlbB from HOU to Waco using embryonic microinjection according to the approach described previously (17). Thus, the same donor and recipient mosquitoes were used to generate the WB1 and WB2 lines. In brief, cytoplasm from each donor embryo was transferred to the posterior of a recipient embryo (60 to 90 min old) by using an IM300 microinjector (Narishige Scientific). After injection, the embryos were incubated at 85% RH and 27°C for 1 h and transferred to wet filter paper. They were then allowed to mature for 5 to 7 d before being hatched. Females (G0) developing from the surviving embryos were isolated and mated with Waco males. After blood-feeding and oviposition, the G0 females were tested for *w*AlbB infection by PCR using the strain-specific primers described below. G1 females were again crossed with Waco males, blood-fed, isolated, and allowed to oviposit. The offspring from the *w*AlbB-positive G1 were selected for the next screen, and this process was repeated until the *w*AlbB maternal transmission rate reached 100%. The *w*AlbB-positive females also assayed for the presence of *w*AlbA by PCR, and none of them tested positive for *w*AlbA infection.

### PCR assay of *Wolbachia* infection

Genomic DNA was extracted from whole bodies, ovaries, or testes of 7-d-old mosquitoes, with 7 to 8 replicates for each treatment, using a Thermo Scientific Phire Animal Tissue Direct PCR Kit (F-140WH). All mosquitoes were reared in 30 × 30 × 30 cm standard cages under controlled condition to ensure collected mosquitoes have similar size. Samples were pretreated in 20 µl dilution buffer with 0.5 µl DNARelease Additive. The reaction mixture contained 10 µl 2X Phire Animal Tissue PCR Buffer, 0.4 µl Phire Hot Start II DNA Polymerase, 0.2 µl of both the forward and reverse primers, and 7.2 µl distilled H2O. The regular PCR conditions were: initial denaturation at 98°C for 6 min, followed by 40 cycles of 5 s at 98°C, 5 s at 56°C, and 45 s at 72°C. qPCR was performed using a QuantiTect SYBR Green PCR Kit (Qiagen) and ABI Detection System ABI Prism 7000 (Applied Biosystems, Foster City, CA, USA). The primers for *w*AlbA, *w*AlbB, and mosquito *rps6* were used as previously described (65). Standard curves were generated for each of the above genes to convert the Ct value for the qPCR into the copy number for each target sequence.

### CI crosses

CI crosses were conducted as previously described (17). Ten virgin males were mated with ten virgin females, with three replicate cages for each cross. A bloodmeal was provided to the females at day 7 post-eclosion. Two days after the bloodmeal, eggs were collected into oviposition cups containing wet filter paper, which was subsequently desiccated for 7 d at 27°C and 80% RH. Eggs were counted and then hatched in water containing 6% m/v bovine liver powder. Larvae were counted at the L2–L3 stage to record the hatch rate.

### Outcrosses to develop WB2 lines with a Mexican or Singaporean genetic background

To introduce the Mexican *Ae. aegypti* genetic background into the WB2 line, we crossed the WB2 line with wild-type mosquitoes collected from the field in Merida, Mexico (AFM) for seven generations (Figs. S2 and S3). During each cross, 100 virgin WB2 females and 100 AFM males were randomly selected. The offspring from each cross were tested for maternal transmission rate. The maternal transmission rates of the outcrossed Mexican WBM line were maintained at 100% during the crosses (Fig. S3). The same procedure was performed to introduce the Singaporean genetic background into WB2, with 100% maternal transmission rates of *w*AlbB maintained at each generation. These outcrossed mosquitoes were subsequently used for the vector competence and mating competitiveness assays described below.

### Vector competence assay

The viruses listed below were used for comparison of *w*AlbB-mediated blocking effects on DENV, CHIKV, and ZIKV: DENV-2 EHIE18944Y13 (KR779784), ZIKV MR766 strain (ATCC), and CHIKV EHIKJ71albY08 (66). After propagation in Vero cells (ATCC CCL-81), the supernatants were mixed with an equal part of swine packed red blood cells. Adenosine triphosphate (ThermoFisher Scientific, USA) was added to the infectious bloodmeal as a phagostimulant at a final concentration of 3 mM. The virus titers used in the infectious bloodmeal were 6.24, 8.74, and 7.74 log10TCID50/ml for CHIKV, ZIKV, and DENV-2, respectively, before mosquito feeding. Two ZIKV lineages, SG(EHI)ZIKV/33164Y17, a South American lineage (GenBank accession no. MF988734) (67); and LP0210Y17, an Asian lineage (not submitted to GenBank) were used to compare *w*AlbB-mediated blocking effects in *Ae. aegypti* on either a Singaporean or Mexican genetic background. Both lineages were isolated from clinical samples in 2017 and had been passaged three times in Vero cells (ATCC, USA) prior to oral infection of the mosquitoes with viral titers of 4.95 and 6.52 log10TCID50/ml for the South American and Asian lineages, respectively. All mosquitoes in Fig. 4 were tested at the same time with the same virus titration assay in randomized designs. One experimental replication was performed for each vector competence assay and there were no blocks in the experiments. The mosquitoes (5 to 7 d old) were fed on a virus-spiked bloodmeal for 45 min. At 6 to 7 and 13 to 14 DPI, the midgut and salivary glands were sampled to measure viral titers. The virus levels were determined using a viral titration assay and expressed as log10TCID50/ml (49).

### Mating competitiveness assay

Fifty AFM females, 50 AFM males and varying numbers of WBM males (0, 50, 250, or 500) were placed in adult cages. Additional cages with either 50 AFM females and 50 WBM males or 50 AFM females and 50 AFM males were set up as the control groups for sterile or fertile mating, respectively. Mosquitoes were allowed to mate for 2 d before bloodfeeding for 20 min. Two days after the bloodfeeding, egg cups were placed in the cages for egg collection. The eggs were then hatched, and the hatch rates were calculated as described for the CI crosses. The egg hatch rate was compared to the expected hatch rate assuming: (i) random mating and equal mating competitiveness between WBM and AFM males, and (ii) complete unidirectional cytoplasmic incompatibility between WBM males and AFM females (8, 68). Male mating competitiveness index was calculated as described previously (8).

### Statistical analysis

Differences between *Wolbachia* density and virus titer were analyzed using a Student’s *t*-test or one-way ANOVA, with *P* values < 0.05 considered significant. Prior to analyses, the normality of the data sets was checked using the D’Agostino and Pearson omnibus normality test. If they were not normally distributed, the Mann–Whittney *U*-test was used for analysis. Virus-negative samples were not included in determining the medians ± 95% CIs and significant difference in viral titers. Differences in the infection rate were evaluated using two-tailed Fisher’s exact test. Variation in virus loads was assessed with a GLM including genetical background, *Wolbachia*, tissue, and their interactions using SAS 9.1. All other analyses were performed in GraphPad Prism v. 7 (GraphPad Software, San Diego, CA, USA).

## Supplementary Material

pgac203_Supplemental_File

## Data Availability

All data are included in the manuscript and/or Supplementary Material.
