# Supplementary material for: Wolbachia wAlbB remains stable in Aedes aegypti over 15 years but exhibits genetic background-dependent variation in virus blocking
Source: PNAS Nexus. 2022 Sep 22;1(4):pgac203. doi: 10.1093/pnasnexus/pgac203 (PMC9802048; doi:10.1093/pnasnexus/pgac203)
Supplement: pgac203_Supplemental_File [file pgac203_supplemental_file.docx]

**Supplemental Figures**


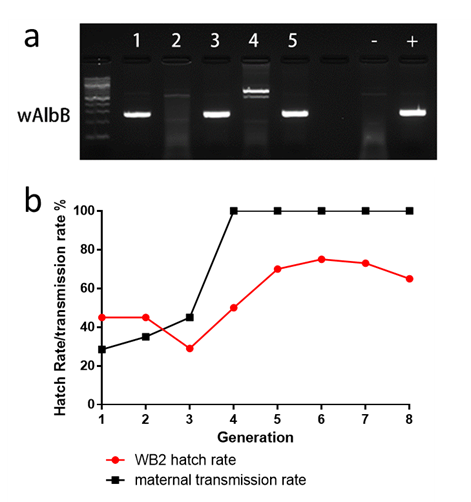


Figure S1. Establishment of the transinfected *Ae. aegypti* WB2 line infected with *w*AlbB. (a) Strain-specific amplification of the *w*AlbB infection by PCR assay of five G0 females that survived the injection in one of three experiments. +, WB1 (positive control), -, wild-type *Ae. aegypti* (negative control). (b) Egg hatch rate and maternal transmission rate of the WB2 line. Egg hatching was calculated as the percentage of eggs hatched divided by the total number of eggs. The transmission rate was calculated as the percentage of *w*AlbB-infected mosquitoes among all the tested mosquitoes.


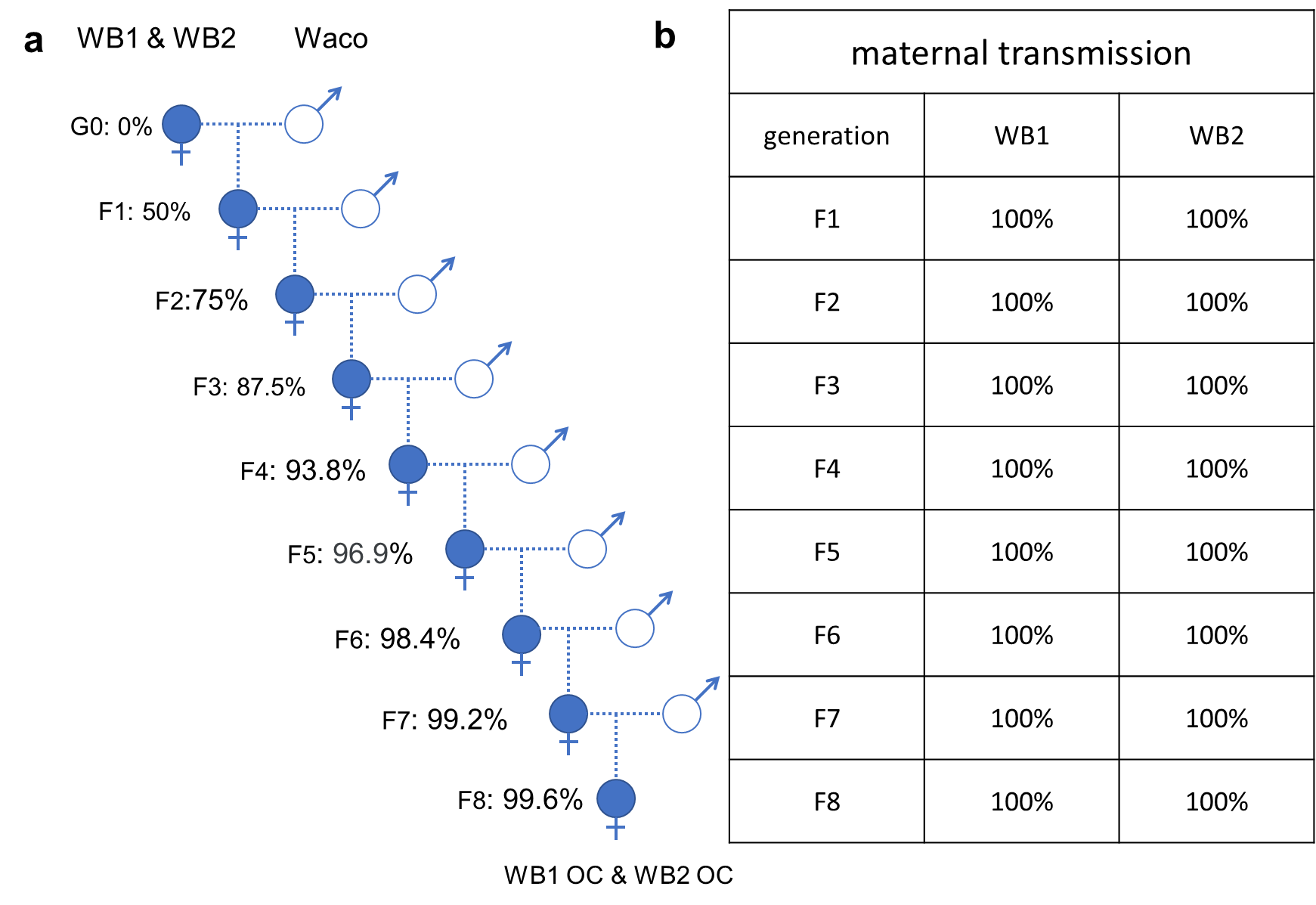


Figure S2. Outcross to establish WB1 and WB2 lines with a homogeneous host genetic background. (a) WB1 and WB2 females were repeatedly outcrossed (OC) with wild-type *Ae. aegypti* Waco males for seven consecutive generations. The numbers for each generation indicate the percentage of the Waco gene background. (b) The maternal transmission rate of WB2 was assayed after each outcross. All the outcrossed generations had a 100% maternal transmission rate. The same strategy was used to generate outcrossed WB2 lines on either the Mexican or the Singaporean genetic background.


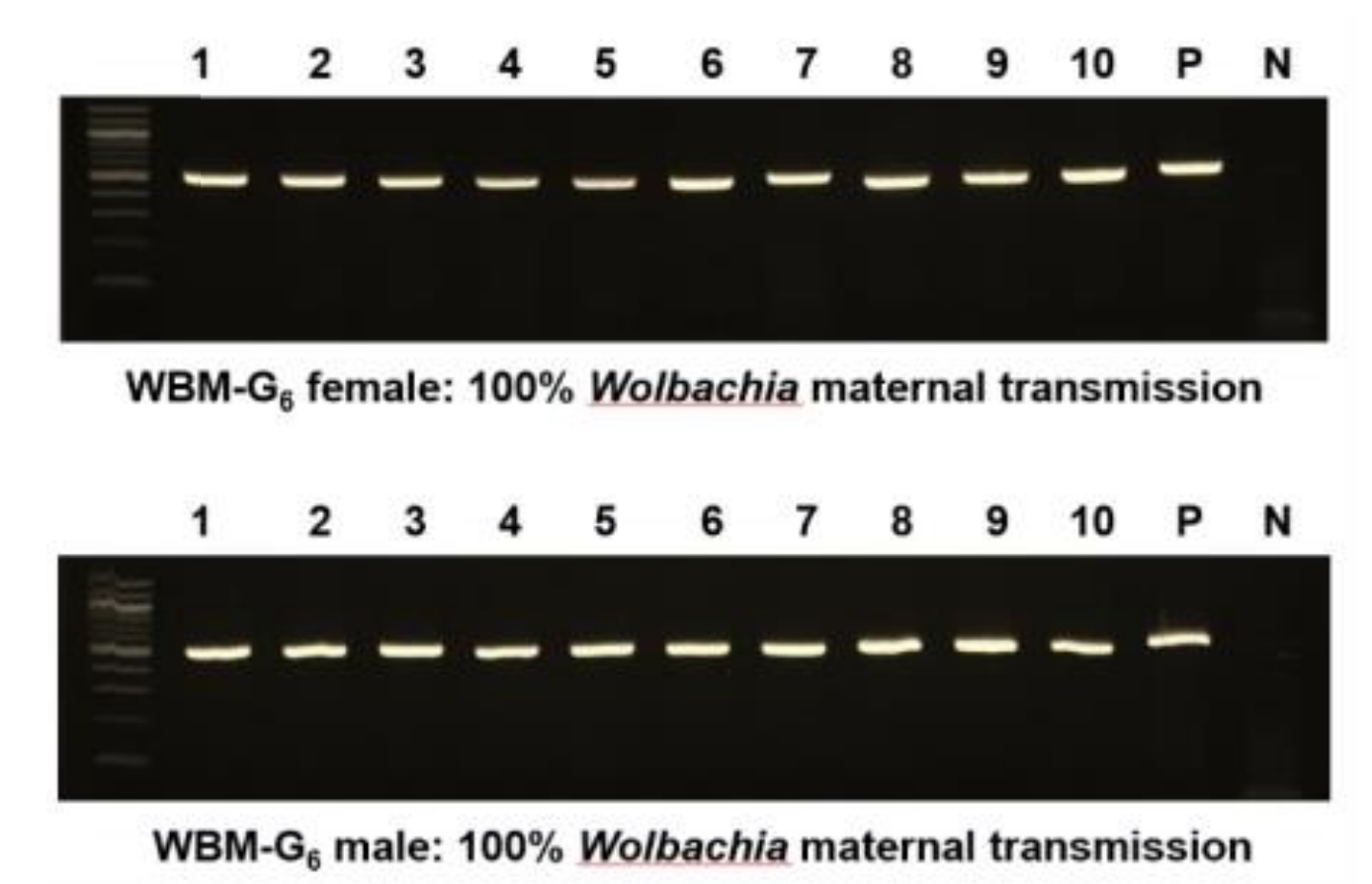


Figure S3. Maternal transmission of *w*AlbB in the WBM line. The outcrossed WB2 line on the Mexican genetic background (WBM) maintained a 100% maternal transmission rate of *w*AlbB. The results for 10 randomly selected females and 10 randomly selected males at G6 are seen in gel electrophoresis for PCR diagnosis. P, positive control. N, negative control.
